# Direct Viewing of Dyslexics’ Compensatory Strategies in Speech in Noise Using Auditory Classification Images

**DOI:** 10.1371/journal.pone.0153781

**Published:** 2016-04-21

**Authors:** Léo Varnet, Fanny Meunier, Gwendoline Trollé, Michel Hoen

**Affiliations:** 1 Lyon Neuroscience Research Center, CNRS UMR 5292, INSERM U1028, Auditory Language Processing (ALP) research group, Lyon, France; 2 Laboratoire sur le Langage le Cerveau et la Cognition, CNRS UMR 5304, Auditory Language Processing (ALP) research group, Lyon, France; 3 Université de Lyon, Université Lyon 1, Lyon, France; UMR8194, FRANCE

## Abstract

A vast majority of dyslexic children exhibit a phonological deficit, particularly noticeable in phonemic identification or discrimination tasks. The gap in performance between dyslexic and normotypical listeners appears to decrease into adulthood, suggesting that some individuals with dyslexia develop compensatory strategies. Some dyslexic adults however remain impaired in more challenging listening situations such as in the presence of background noise. This paper addresses the question of the compensatory strategies employed, using the recently developed Auditory Classification Image (ACI) methodology. The results of 18 dyslexics taking part in a phoneme categorization task in noise were compared with those of 18 normotypical age-matched controls. By fitting a penalized Generalized Linear Model on the data of each participant, we obtained his/her ACI, a map of the time-frequency regions he/she relied on to perform the task. Even though dyslexics performed significantly less well than controls, we were unable to detect a robust difference between the mean ACIs of the two groups. This is partly due to the considerable heterogeneity in listening strategies among a subgroup of 7 low-performing dyslexics, as confirmed by a complementary analysis. When excluding these participants to restrict our comparison to the 11 dyslexics performing as well as their average-reading peers, we found a significant difference in the F3 onset of the first syllable, and a tendency of difference on the F4 onset, suggesting that these listeners can compensate for their deficit by relying upon additional allophonic cues.

## Introduction

Developmental dyslexia is an extensively researched and documented learning disability, and one of the most common causes of reading difficulties, affecting about 5%-10% of school-age children and persisting in adulthood. It is characterized by reading performances well below the normal range for given age groups and IQ levels, and not explained by sensory deficits or insufficient scholarship only. This concise definition contrasts with the heterogeneity of associated cognitive impairments, which are observed in reading tasks [1], but also speech comprehension [2], auditory processing of rapid sounds [3], visual tasks [4], and even postural tests [5].

Although the causes of developmental dyslexia remain opaque, it is acknowledged that a vast majority (75% to 100%) of dyslexic individuals show a phonological deficit, noticeable in tasks involving phonological awareness (e.g. spoonerism), verbal short-term memory (e.g. non-word repetition) or lexical retrieval (e.g. rapid automatic naming tasks) [6–8]. However, the exact nature of this impairment is still debated. Some authors have proposed that it may result from an impairment in the access to phonological representations [9–11], from an abnormal auditory sampling [12,13], or from underspecified [14] or overspecified [15,16] phonological representations, leading in all cases to a blurring of boundaries between phonological categories. One major difficulty in testing these hypotheses arises from the fact that dyslexics do not form a homogeneous population, showing very different patterns of errors. Thus dyslexia is often divided into subtypes, possibly originating from deficits at various stages of the comprehension system [1,17]. The pattern of speech deficits in developmental dyslexia has been usually investigated by comparing a group of participants with dyslexia to a group of controls matched for chronological age or for reading level. However, given the variety of subtypes, group differences may mask a wide variability in behavioral responses of dyslexic participants. Therefore, some authors have also employed individual deviance analyses to assess abnormal performances at the individual level [7,8,18].

With experience, dyslexics often develop compensatory strategies for speech recognition, giving the misleading impression that dyslexia disappears with time. Indeed, they usually demonstrate no or weak deficits for speech perception in quiet, probably because they capitalize on the very redundant nature of speech. However, difficulties are still reported under more challenging conditions, such as in the presence of background noise. By the age of 8, children with dyslexia perform as well as matched normal readers in a speech-in-quiet task, but they still experience difficulties understanding noisy speech [19,20]. This deficit can in turn become particularly disabling to achieve normal school progress in the context of a noisy classroom. Along the same line, it is now well acknowledged that dyslexics [21,22] and children with a familial history of dyslexia [18] are generally more affected by the presence of strong background noise, compared to matched controls without learning disabilities. By contrast, tone-in-noise detection deficits are weaker and found only in specific subgroups [8,23] suggesting that speech-in-noise difficulties stem from a phonological problem rather than an auditory problem. It may have roots in poorer encoding of speech sounds at the subcortical level, as evidenced by speech auditory brainstem responses [24–27]. The impaired robustness of the representation of speech in the presence of noise has even been proposed as a core deficit of dyslexia. Indeed, behavioral measures of speech-in-noise comprehension predict reading performances better than other cognitive, auditory or attentional measures [20]

Some authors have nevertheless highlighted that the speech-in-noise impairment is not always observed [28], and highly dependent on the type of background and the listening configuration used [7,21], dyslexics even showing better release from masking than normal reading controls in some cases [20,21]. This again suggests that alternative processing strategies may be employed to compensate for the deficit of speech recognition in noise. For instance, concurrent babbles are particularly deleterious to dyslexic’s comprehension, when information about sound-stream localization is not available, but not in more natural conditions, when speech and noise originate from two separate sources [21]. In the latter case, however, dyslexics show a right hemisphere over-activation, suggesting a reallocation of neural resources [22]. In the same vein, two studies demonstrated that dyslexics [29] or children at risk for dyslexia [30] with no apparent behavioral deficits in a phoneme categorization task nevertheless show heightened sensitivity to non-linguistic information in their neural responses. Therefore, even when they behaviorally compensate for their phonological difficulties, dyslexics still show neurophysiological evidence of a less efficient processing of phonemes.

The present experiment aimed at exploring the listening strategies employed by dyslexic individuals for phoneme categorization. Here we chose to test the distinction between places of articulation in stop consonants, which has been shown to be particularly impacted in dyslexics [20,31]. As we mentioned earlier, the addition of a sufficient amount of background noise is necessary to reveal their difficulties, which are otherwise compensated. In this context, the Auditory Classification Image (ACI) method seems perfectly adapted as it relies on a phoneme categorization task in noise to derive individual maps of participant’s listening strategies.

The ACI method [32–34] has been developed as an auditory version of the Classification Image method, which aims at revealing the primitives used in various visual detection or categorization [35–38] and more broadly the strategies used to make decisions in forced-choice tasks [39–43]. Another way of seeing the ACI is as a behavioral spectrotemporal receptive field, derived from the responses of a participant instead of those of auditory neurons [44–46]. This technique relies on a forced-choice phoneme categorization task performed in noise. The idea here is to capitalize on the masking noise to predict the responses of the listener on a trial-by-trial basis: by training a statistical model on the categorization data we can uncover how a specific noise configuration misleads the participant towards one particular phoneme. The result is a spectrotemporal perceptual map showing the time-frequency regions where the presence of energy influences the phonemic decision. Therefore this method allows us to visualize which parts of the speech stimuli serve as auditory cues for phoneme categorization.

The ACI method has already been successfully used with normal-hearing participants performing a /da/-/ga/ discrimination in context /al/ or /aʁ/ [33]. We have identified 3 acoustic cues mainly involved in this task: the F1, F2 and F3 formant onsets. This estimate was precise enough to allow the comparison between different groups of participants. In a follow-up experiment, we asked a group of musician experts to perform the same categorization task [34]. As expected, professional musicians showed a better resistance to noise than non-musicians. In order to determine if these improved performances were driven by a refinement of their listening strategies, musicians’ ACIs were calculated to examine potential changes in the acoustic cues used. Contrary to what may be initially assumed, the ACIs of the two groups were qualitatively similar, suggesting that all participants followed the same strategy. However, musicians relied more heavily on the two main acoustic cues. This result offers a direct proof that the training of purely auditory skills percolates to speech comprehension by a reweighting of time-frequency regions used as speech categorization cues. The analysis of prediction rates of ACIs models for musicians and non-musicians revealed that the second group responded more consistently to speech stimuli.

The present experiment followed the same procedure, with a group of participants with dyslexia compared to a group of normal reading participants. The purpose was to determine if there was any significant group difference in the weighting of the acoustic cues that may reveal the phonological deficit in dyslexics or the compensatory strategies they developed. We reasoned here that if their representations of phonemes were under- or overspecified in a consistent way, one should observe large dissimilarities in their ACI, on average, compared to those of normal-hearing controls.

## Material and Methods

The study was approved by the Comité d'évaluation éthique de l'Inserm / Institutional Review Board (IORG0003254, FWA00005831, IRB00003888). Participants provided written informed consent before participating in the experiment.

### Participants

Twenty French-native volunteers with dyslexia took part in this study. Participants were informed about the experimental procedure used before they provided written consent, and they received a financial compensation for their participation (100€). They all had prior diagnosis of dyslexia from a psychologist. Participants had the option of ending the experiment at any time, but none of them did. From these original recordings, 2 participants had to be rejected from the dataset due to excessively low performances. The analyses reported in the following are thus based on 18 recordings (11 females, age 22.8 years ± 6.5 years S.D.).

Eighteen typical readers were selected from a previous study [33] to match the dyslexic group in age, nonverbal IQ and handedness (12 females, 22.8 years ± 4.5 years S.D.). All participants included in the present study had normal hearing (audiometric pure-tone threshold ≤ 20 dB on the 125 Hz– 8000 Hz range for both ears), and reported no history of neurological disorders.

### Cognitive and phonological tests

Attention capacities were evaluated using the Attention Network Test (ANT) [47]. Nonverbal IQ was assessed by the Raven’s Standard Progressive Matrices. All participants obtained normal scores above the 50^th^ percentile of their age category (corresponding to a score of at least 42/60). Literacy and phonological skills were assessed by the ECLA-16+ [48]. This battery includes the French-language standardized L’Alouette Reading Test [49], phonological awareness tests (phoneme deletion and spoonerism), reading and spelling tests, and working memory tests (digit span, backward/forward). All results are reported Table 1. The details of the results can be found at https://zenodo.org/record/29239.

**Table 1:** Summary of the characteristics of the dyslexic and normal-reading groups.

	Dyslexic group	Normal-hearing group	t-test
N	18	18	
Gender (f/m)	11/9	12/8	
Age	22.83 (± 6.52 S.D.)	22.83 (± 4.48 S.D.)	p = 1
Handedness (Edinburgh test)	73.33 (± 30.48 S.D.)	62.78 (± 55.17 S.D.)	p = 0.469
Raven’s Standard Progressive Matrices (score /60)	49.67 (± 4,40 S.D.)	50.83 (± 4,12 S.D.)	p = 0.436
Reading age in months (L'Alouette MCLM)	124.06 (± 26,54 S.D.)	186.78 (± 24,7 S.D.)	p = 3.10^−8^***
Reading tests			
regular words (score /20)	19.11 (± 0,74 S.D.)	19.72 (± 0,45 S.D.)	p = 6.10^−3^**
regular words (time in s)	21.39 (± 8,43 S.D.)	11.94 (± 1,99 S.D.)	p = 7.10^−5^***
irregular words (score /20)	18.06 (± 1,93 S.D.)	19.11 (± 0,81 S.D.)	p = 0.045*
irregular words (time in s)	20.22 (± 7,64 S.D.)	12.17 (± 2,11 S.D.)	p = 1.10^−4^***
pseudowords (score /20)	17.17 (± 3,27 S.D.)	18.78 (± 2,27 S.D.)	p = 0.105
pseudowords (time in s)	35.94 (± 14,98 S.D.)	18.5 (± 3,24 S.D.)	p = 4.10^−5^***
Spelling tests			
sentences: orthography (score /10)	6.56 (± 1,86 S.D.)	9.28 (± 0,65 S.D.)	p = 2.10^−6^***
sentences: grammar (s/10)	6.17 (± 2,39 S.D.)	8.67 (± 0,67 S.D.)	p = 2.10^−4^***
regular words (score /10)	7.17 (± 1,34 S.D.)	9 (± 0,75 S.D.)	p = 2.10^−5^***
regular words (time in s)	48.39 (± 6,30 S.D.)	38.72 (± 5,64 S.D.)	p = 4.10^−5^***
irregular words (score /10)	4.39 (± 1,92 S.D.)	7.833 (± 1,30 S.D.)	p = 5.10^−7^***
irregular words (time in s)	53.33 (± 10,96 S.D.)	42.06 (± 8,98 S.D.)	p = 2.10^−3^**
pseudowords (score /10)	7.06 (± 1,54 S.D.)	8.78 (± 1,84 S.D.)	p = 5.10^−3^**
pseudowords (time in s)	59.44 (± 14,80 S.D.)	45.22 (± 4,88 S.D.)	p = 6.10^−4^***
Phonological awareness tests			
Phoneme deletion (score /10)	6.5 (± 2,22 S.D.)	9.22 (± 1,90 S.D.)	p = 5.10^−4^***
Phoneme deletion (time in s)	45.78 (± 11,14 S.D.)	27.78 (± 6,20 S.D.)	p = 1.10^−6^***
Spoonerism (score /20)	15.5 (± 4,04 S.D.)	19.22 (± 0,97 S.D.)	p = 7.10^−4^***
Spoonerism (time in s)	141.72 (± 55,71 S.D.)	69.22 (± 19,68 S.D.)	p = 1.10^−5^***
Memory span tests			
Pseudowords repetition (score /20)	18.72 (± 1,24 S.D.)	19.5 (± 0,69 S.D.)	p = 0.030*
Forward digit	6.17 (± 1.01 S.D.)	7.22 (± 0.98 S.D.)	p = 3.10^−3^**
Backward digit	4.56 (± 1.21 S.D.)	6.11 (± 1.20 S.D.)	p = 6.10^−4^***
ANT			
alerting effect	32.89 (± 26.28 S.D.)	33.06 (± 21.76 S.D.)	p = 0.984
orienting effect	53.00 (± 15.38 S.D.)	38.83 (± 22.46 S.D.)	p = 0.039*
conflict effect	155.39 (± 42.23 S.D.)	130.44 (± 36.82 S.D.)	p = 0.075

Statistical significance is indicated with *** for p<0.001, ** for p<0.01 and * for p<0.05.

### Stimuli

Targets were borrowed from a previous study [33]. They consisted in 4 /aCCa/ nonwords with a continuant consonant (/l/ or /ʁ/) followed by a stop consonant (/d/ or /g/). All sounds were recorded by a male speaker and digitized at a sampling rate of 48 kHz (16-bit). Targets were equated both in total length (680 ms) and duration of the 1^st^ syllable (328 ms) by cutting of the end of the syllables when necessary. The resulting audio.wav files can be downloaded at https://zenodo.org/record/12300.

For each participant, a set of 10,000 white noise instances of same duration as the targets were generated and stored before the beginning of the experiment. These files can be found at the addresses https://zenodo.org/record/12374 and https://zenodo.org/record/19102.

### Experimental procedure

Participants sat in an acoustically isolated chamber in front of video monitor where they read instructions for the experiment. They wore Sennheiser’s HD 448 headphones. On a given trial, they were presented with one of the four possible targets superimposed with additive white noise. The SNR was adapted from one trial to the next based on the performance level using a 3-down 1-up staircase procedure to target the 79% correct point [50]. All stimuli were power-normalized and presented at each participant’s most comfortable sound level.

The task of the participant was to identify the last syllable of the stimulus as /da/ or /ga/, independently of the preceding consonantal context, and to respond as quickly as possible by a button press. The response to trial *i* is denoted *r*_*i*_ (0 for ‘da’ and 1 for ‘ga’). Participants were allowed to play the stimulus as many times as needed, however they nearly always respond after the first listening. The experiment was divided into 20 sessions of 500 trials each, separated with breaks and completed over 4 days. The total length of the experiment (10,000 stimuli plus cognitive and phonological tests) was approximately 4 hours. Data from all participants are available at https://zenodo.org/record/12303 and https://zenodo.org/record/19129.

### Auditory Classification Images

We previously described a method for deriving individual Auditory Classification Images (ACIs) for a closed-set categorization task in noise [33,34]. This method is inspired from similar works in the visual domain [35–37]. The ACI calculation has three steps. First a cochleogram is generated for each sound stimulus (54 frequency steps spaced quasi-logarithmically and 81 time steps). For each trial *i*, time-frequency bins of the cochleogram are treated as a vector of predictors and denoted *S*_*i*_. Second, data are randomly divided into 10 sets of 1000 trials that will be assigned to the test set or training set during cross-validation. ACIs are derived using a regularized logit regression between the physical properties of the stimulus *S*_*i*_ and the corresponding response of the participant *r*_*i*_ [42]. The resulting vector of parameters *β* can be thus seen as a weighting function of the cochleogram. Here we implemented a smoothing constraint penalizing abrupt variations in the ACI, with a level of smoothing *λ* determined by a 10-fold cross-validation [51]. For each value of *λ*, 10 ACIs are estimated, by each time putting aside one set of 1000 trials and using the remaining 9000 trials as training set. Once the ACIs are obtained, their generalizability is measured in terms of cross-validated deviance (CVD) and cross-validation rate (CVR) by predicting data that were not used in the estimation (test set). During this process, proportions of correctly and incorrectly categorized trials are equated in each training set and each test set to make sure that the performance level of the participant does not impact the estimation or evaluation of the models. Third, the level of smoothing *λ* yielding the lowest mean CVD over all participants is selected and ACIs are re-computed on the complete datasets (10,000 trials) using this value of lambda.

### Statistical analyses

Participant’s listening strategies were compared on a group basis. Two aspects were investigated: whether the weighting of time-frequency information was similar between dyslexics and control participants, and whether the individual listening strategies were more heterogeneous in one group. Correspondingly, two types of statistical analyses were performed.

Firstly, we used a cluster-based non parametric test to know if there was a significant difference between the ACIs of the two groups. This statistical test is appropriate when dealing with highly dimensional data where the location of the potential effect is unknown [52]. The general procedure is as follows: Clusters of adjacent pixels weighted significantly differently between two conditions are identified by a running t-test. Then a permutation test (5000 randomizations) is performed to determine which of them were unlikely to have occurred by chance. Applied to ACIs, this allows us to detect fine differences in the template of weights between two groups or two conditions [33]. This picture was completed by a classic ROI analysis. As in [34], ROIs contours were defined as clusters of at least 7 adjacent time-frequency bins identified as significant in a running t-test (p<10^−10^). Then the mean weights in each set were separately compared between the two groups with another t-test.

Secondly, the generalizability of each participant’s ACI was evaluated by measuring how well it can predict the responses of each other participants from both groups. For this purpose, each model was characterized not only by its 10-fold CVD and CVR derived during the estimation process (“auto-prediction deviance” and “auto-prediction rate”) but also 2N-1 between-subject CVDs (“cross-prediction deviances”). Here, the predictive power is assessed in the same way as before, except that the test set is now taken from another participant’s data. A measure of the specificity of a listener’s strategy can be obtained by taking the difference between his/her auto-prediction deviance and the mean cross-prediction deviance of his/her data produced by ACIs of the other participants. This value is high when the responses are better predicted by his/her own ACI than those of the other.

## Results

### Cognitive and phonological tests

As a group, dyslexic participants enrolled in this study performed significantly lower than control participants (p < .05) on all tests but Raven’s (p = 0.436) and pseudo-words reading (p = 0.105) tests (see Table 1).

To confirm the reading impairment on an individual basis, we performed an “individual deviance analysis” as described in [8] (see also [7]). This two-step procedure considers a performance as deviant when it exceeds 1.65 S.D. from the mean of a given distribution (the fifth percentile of the normal distribution). For each measured characteristics, the mean and standard deviation were calculated on the control group. Any abnormal performances deviating of more than 1.65 S.D. from the mean were then removed from the group and the mean and standard deviation were recomputed. Finally, deviant dyslexic participants were identified on the basis of these new values, with the same 1.65 S.D. threshold. According to this criterion, all dyslexic participants deviated from the normal range of performance in at least 12 of the 26 characteristics, confirming the diagnosis.

### Performances in the main experiment

As expected, dyslexic participants were poorer than normal hearing participants on the main phoneme categorization-in-noise experiment. Although both groups obtained similar correct response rates thanks to the adaptive SNR algorithm (dyslexics: 78.8% ± 0.4% S.D.; controls: 78.8% ± 0.4% S.D.; t(34) = 0.42; p = 0.67), and similar sensitivity (d`) as defined in signal detection theory (dyslexics: 1.64 ± 0.04 S.D.; controls: 1.65 ± 0.06 S.D.; t(34) = 0.49; p = 0.62), dyslexics performed the task at a +1.31 dB SNR higher than normotypical controls on average (dyslexics: - 10.59 dB ± 1.87 dB S.D.; controls: -11.90 dB ± 1.07 dB SNR; t(34) = 2.58; p = 0.014). Furthermore, dyslexic participants responded slower than controls (dyslexics: 1.38 s ± 1.87 s S.D.; controls: 1.28 s ± 0.12 s S.D.; t(34) = 2.38; p = 0.023).

**Fig 1:**
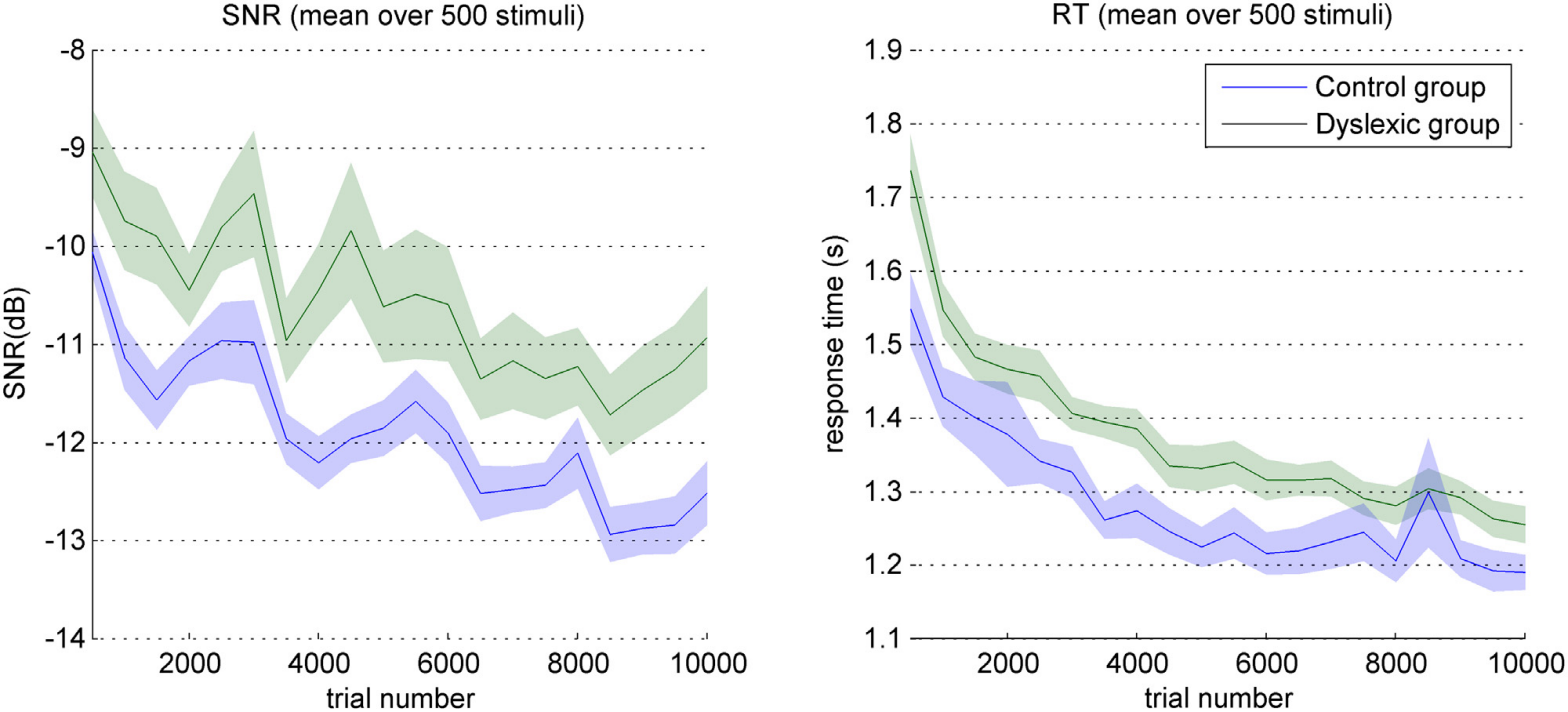
Evolution of performances over the course of the experiment. Mean SNR (left) and mean RT (right) by sessions of 500 trials, for the two groups. Shaded regions denote s.e.m. over participants.

To assess the extent of any learning effect we carried out two separate 2-way repeated-measures analyses of variance (ANOVAs) on the SNR and response time data. The results show that participants’ performances improved over the course of the experiment (Fig 1): we obtained significant effects of session number (F(19,34) = 16.63; p<0.001) and group (F(1,34) = 6.68; p<0.05) on SNR, and significant effects of session number (F(19,34) = 33.42; p<0.001) and group (F(1,34) = 5.65; p<0.05) on RT. In both cases interaction effects were not significant, suggesting that the magnitude of learning effect is equivalent in the two groups.

### ACIs

**Fig 2:**
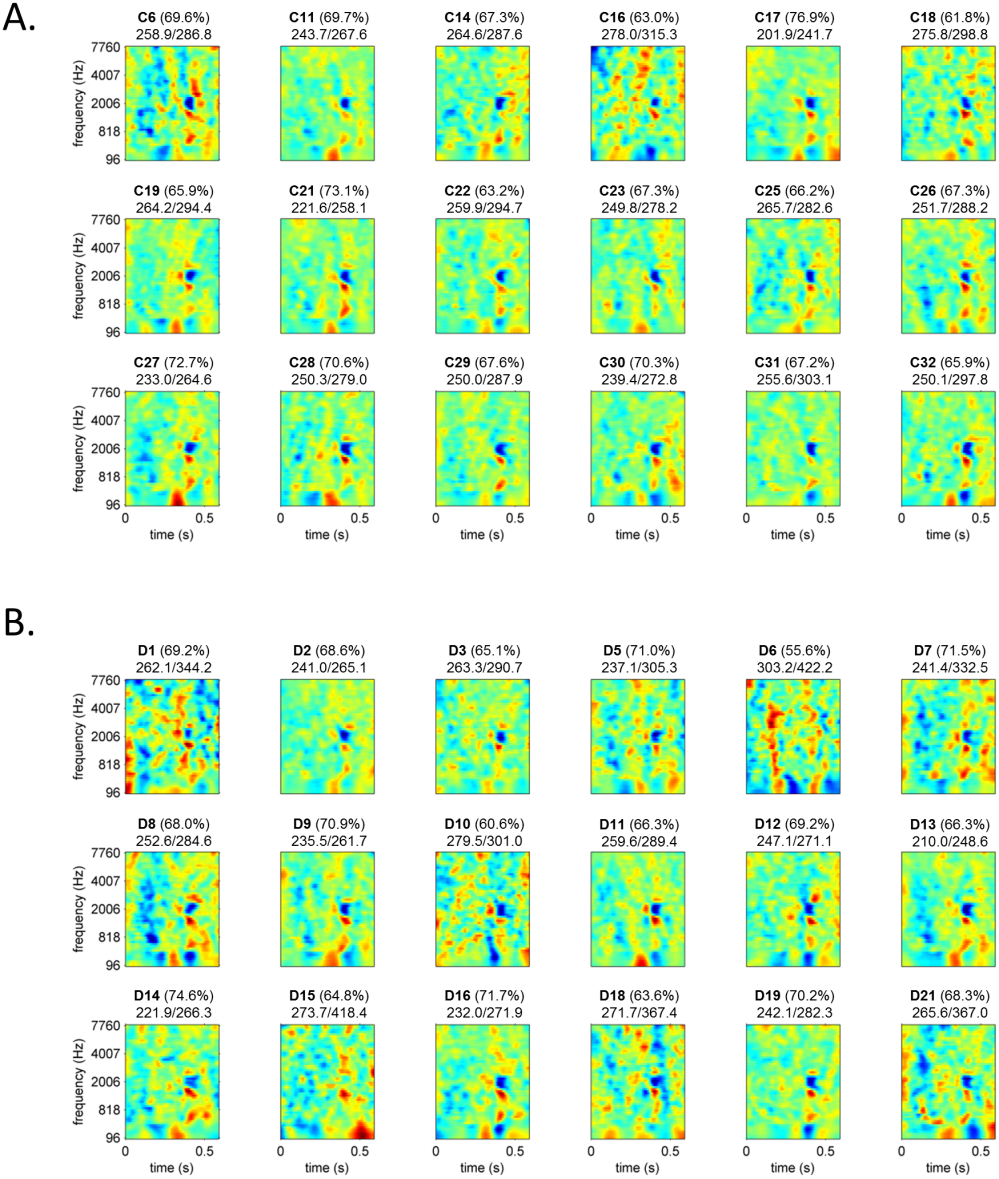
Individual ACIs for all control (A.) and dyslexic (B.) participants. For each ACI the auto-prediction rate is given (in brackets), followed by the auto- and cross- prediction deviances. Positive weights (in red) are time-frequency bins where the presence of noise increases the probability that the listener gives the response “da” whereas negative weights (in blue) are time-frequency bins where the presence of noise increases the probability that the listener gives the response “ga”.

ACIs were calculated for each dyslexic participant and compared with those of their average reader peers. Here, positive weights are time-frequency bins where the presence of noise increases the probability that the listener gives the response “da”, whereas negative weights are time-frequency bins where the presence of noise increases the probability that the listener gives the response “ga”. Individual images are shown in Fig 2. For each participant, the quality of the ACI was assessed by its cross-validation rate (auto-prediction rate), ranging from 55.5% to 76.9%.

**Fig 3:**
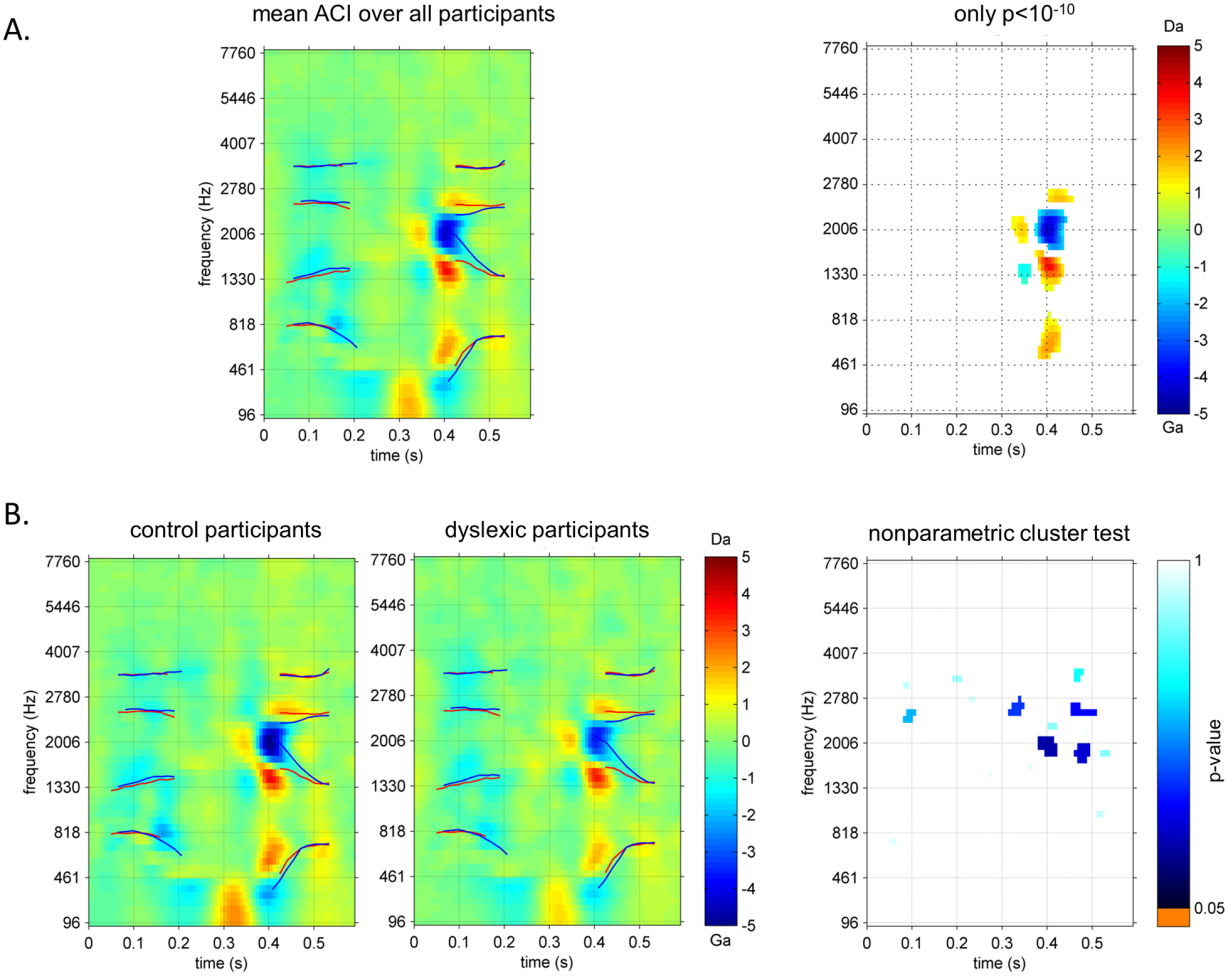
Diagram of the group-analysis of ACIs used in this study. A. mean ACI over all participants (left) and same ACI with all non-significant pixels (p>10^−10^) plotted in white (right panel) defining the regions used in the ROI analysis. B. mean ACIs for the control (left panel) and dyslexic (center panel) groups and output of the cluster-based non-parametric test (right panel). In the ACIs, lines correspond to mean formant trajectories for /alda/ and /aʁda/ (red) and for /alga/ and /aʁga/ (blue).

Generally, they all shared a similar pattern of weight despite some inter-individual variability. This common pattern becomes clearer when considering the mean ACI over all participants (Fig 3A). As already noticed in a previous study [34], the most consistently weighted areas (p<10^−10^) are the onsets of the F1 F2 and F3 in the second syllable (Fig 3A).

Our first aim being the comparison of the two groups, we averaged separately the dyslexics’ and controls’ ACIs to obtain two group-ACIs (Fig 3B). The striking similarity between them is corroborated by a cluster-based non-parametric test eliciting no significant differences (p>0.05 for all clusters, Fig 3B).

In order to confirm this result, a less-conservative ROI analysis was performed in the significant regions identified on the pooled images of the two groups, in the previous step. In each of the 6 ROIs, the weights were averaged for each participant, and then compared using a non-paired t-test. The central negative region appeared to be significantly less weighted by dyslexic than control participants (p = 0.009), while all other differences were non-significant. The characteristics of the ROIs are summarized in Table 2.

**Table 2:** Summary of the characteristics of all sets of weights, sorted by bias and latency.

Set	size (pxl)	Centroid		Extent		Correspondence with formants	Bias towards	Set weights				
		t (ms)	f (Hz)	t (ms)	f (Hz)			C group (mean)	C group (SD)	D group (mean)	D group (SD)	t-test (D vs. C)
#1	16	340	1967	29	329	onset F2/F3, 2^nd^ syllable	‘da’	0.0049	0.0021	0.0051	0.0023	0.79
#2	34	406	1380	58	425	onset F2, 2^nd^ syllable	‘da’	0.0167	0.0049	0.0161	0.0044	0.73
#3	33	406	665	44	366	onset F1, 2^nd^ syllable	‘da’	0.0137	0.0050	0.0103	0.0054	0.06
#4	14	427	2557	51	138	onset F3, 2^nd^ syllable	‘da’	0.0051	0.0014	0.0047	0.0019	0.44
#5	10	350	1347	22	166	onset F2, 2^nd^ syllable	‘ga’	-0.0028	0.0010	-0.0024	0.0015	0.27
#6	48	409	1977	66	549	onset F2/F3, 2^nd^ syllable	‘ga’	-0.0358	0.0061	-0.0263	0.0132	0.009**

Statistical significance is indicated with ** (p<0.01).

**Fig 4:**
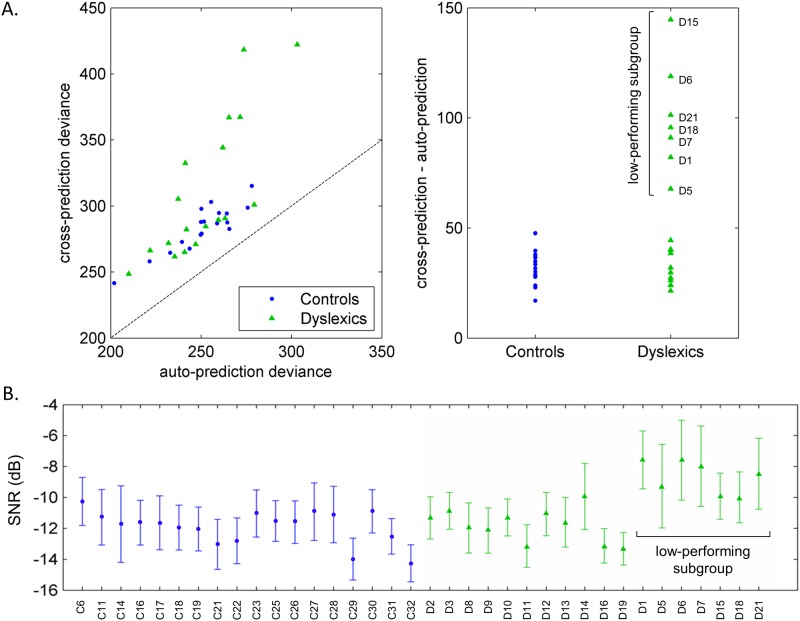
Individual strategies and performances in the task. A. Auto- and cross-predictions for all participants. Left panel: cross-prediction deviance as a function of auto-prediction deviance. The dotted line indicates the cross-prediction = auto-prediction line. Right panel: difference between cross- and auto-prediction deviances for the two groups. B. SNR threshold and confidence intervals for each listener. From these two representations, 7 dyslexics clearly stand out as a low-performing subgroup.

The lack of difference between the results of the two groups could be due to the inter-individual variability observed in the dyslexic group (Fig 2). Furthermore, SNR acts as a confounding variable because dyslexics performed the task in significantly lower levels of background noise. This encouraged us to look more closely at potential individual strategies which may have obscured the picture. To this end, we measured how well each participant’s data are predicted by his/her own ACI, one the one hand, and by those of each other participant in average, on the other. This resulted in two measures, respectively the auto- and cross-prediction deviances. Auto-prediction deviance (error when predicting new data from one listener with his own ACI) did not differ significantly between the two groups (dyslexics: 252.2 ± 22.5 S.D.; controls: 250.8 ± 18.6 S.D.; t(34) = 0.21; p = 0.84), but cross-prediction deviance (error when predicting new data from one listener with ACIs of the other participants) is significantly higher for dyslexic participants (dyslexics: 310.5 ± 53.1 S.D.; controls: 283.3 ± 17.7 S.D.; t(34) = 2.06; p = 0.049). It is clear from the plot of the cross-prediction deviance against the auto-prediction deviance (Fig 4A) that the first is always higher than the second. This stems from the fact that there is a portion of each participant’s data that can be accurately predicted by his own ACI only, which captures some particularities of the strategy.

Our main interest was therefore on the difference between these two measures, viewed here as an indicator of the “specificity” of the listener’s strategy (Fig 4A). On average, the results were dissimilar for the two groups (dyslexics: 58.3 ± 38.0 S.D.; controls: 32.5 ± 8.2 S.D.; t(34) = 2.81; p = 0.008). This difference was mainly due to a subgroup of 7 dyslexic participants with high values (participants D1, D5, D6, D7, D15, D18 and D21), the others being in the normal range. The specificity measure is strongly correlated with the mean SNR at which participants performed the task (r(34) = -0.63, p<0.001), even when restricting the calculus to dyslexics only to avoid any group effect (r(16) = -0.72, p<0.001). Within the control group, the opposite correlation is observed (r(16) = 0.56, p = 0.01); however it should be noted that the variability of specificity in this group is very limited, compared to the dyslexic group. As a result, the 7 dyslexics in the subgroup also stand out in terms of individual SNR thresholds, as can be seen from Fig 4B.

**Fig 5:**
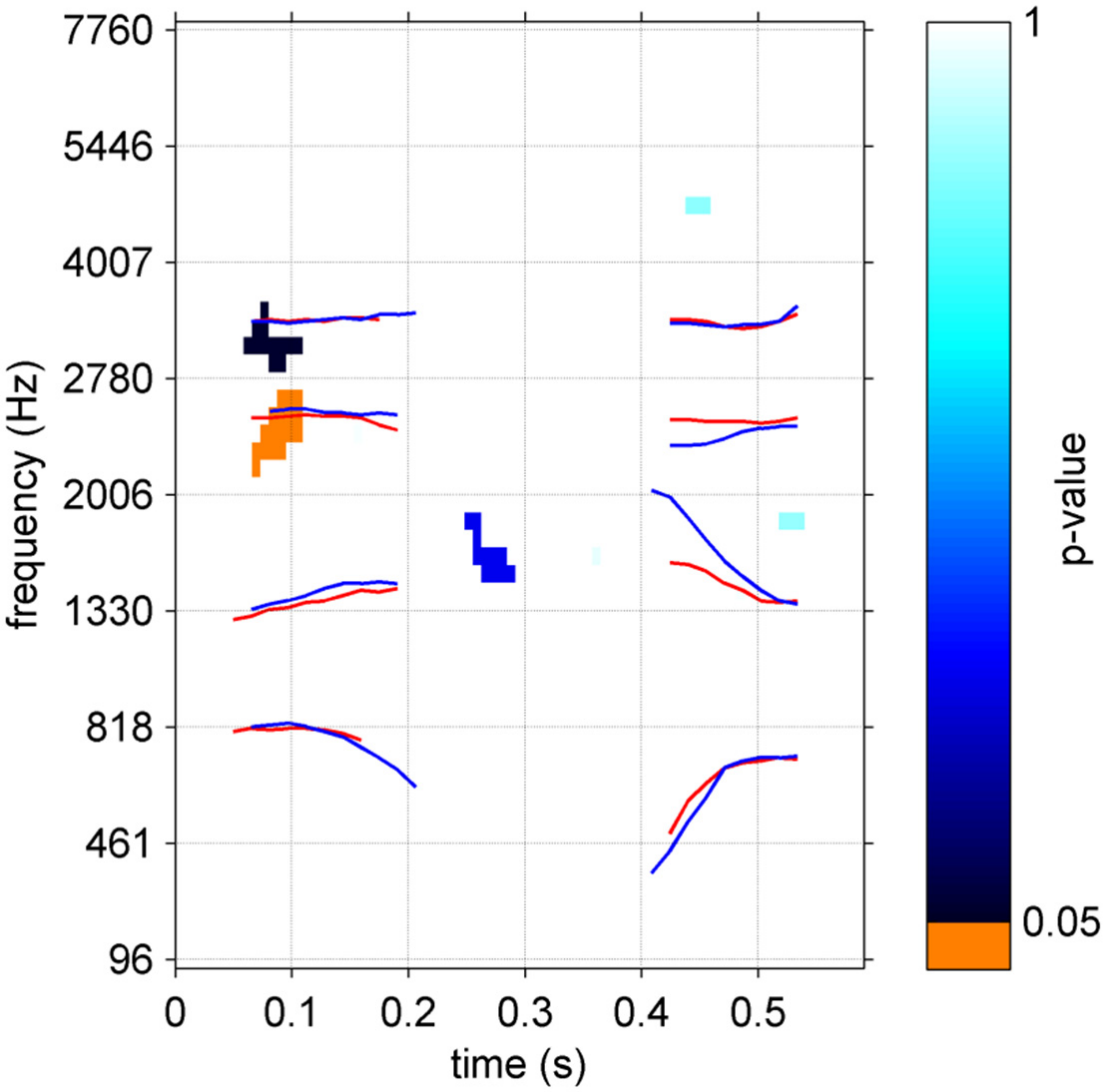
Comparison between the dyslexic and control subgroups matched in SNR (N = 11). Output of the cluster-based non-parametric test. Lines correspond to mean formant trajectories for /alda/ and /aʁda/ (red) and for /alga/ and /aʁga/ (blue).

As can be seen from Fig 4, 11 listeners among the dyslexic group used comparable strategies and obtained performances in the normal range. One explanation could be that they somehow compensate for their speech-in-noise deficit. To reveal the mechanisms by which these participants were able to reach the same SNR as controls, 7 control participants were randomly discarded to result in two subgroups (N = 11) of equivalent SNR (dyslexics: -11.80 ± 1.07 S.D.; controls: -11.38 ± 0.57 S.D.; t(34) = 1.14; p = 0.27). Their characteristics are reported as supplementary data (S1 Table). A cluster-based comparison of the ACIs of the two subgroups was performed, revealing a significant cluster on the onset of F3 in the first syllable (p = 0.02) and a cluster showing a tendency on the onset of F4 (p = 0.061) (Fig 5).

## Discussion

Unlike previous studies directly contrasting the performances of dyslexic and normotypical participants in different verbal or non-verbal tasks, this work focused on the estimation and comparison of listening strategies in the two groups. For this purpose we used the recently developed ACI technique, a psychophysical tool that has proven to be efficient for identifying the acoustic cues used in a phoneme categorization task in noise. This offered us an insight into the roots of the phonological impairment in dyslexia and enabled us to visualize the compensatory strategies that might be developed to overcome these difficulties.

Here the task was a simple /da/-/ga/ discrimination with two phonetic contexts, /al/ or /aʁ/. The noise level was adapted online on an individual basis to target the 79% correct point. Each participant completed 10,000 trials and special care was taken to ensure that they were not subject to fatigue of weariness. Data from one group of N = 18 participants with a prior history of developmental dyslexia, and from a control group of N = 18 average readers matched in age, nonverbal IQ and handedness, were analyzed.

As expected, dyslexic participants enrolled in this study obtained performances well below the normal range in the phonological tasks, as a group (see Table 1) but also on an individual basis as indicated by a deviance analysis. This, combined with their normal scores in the cognitive tests and their significant deficit in attention, confirmed the diagnosis of dyslexia. Coherently, the results of the main experiment clearly show that dyslexics are less efficient in their processing of the stimuli: compared to normo-typical participants, dyslexic listeners performed the task with a gap of 1.31 dB SNR and a delay of 100 ms for the same correct response rate (Fig 1). Our first aim in this study was to explore the group-ACIs to identify correlates of this behavioral deficit in the acoustic cues used.

ACIs estimated for all participants are relatively coherent, resulting in a well-defined average pattern of weights in the overall ACI (Fig 3). Two critical time-frequency regions, composed of highly positive (red) or negative (blue) weights, stand out clearly against the non-significant white background (threshold arbitrarily set to p>10^−10^). They are both located around 400 ms, i.e. approximately at the beginning of the 2^nd^ syllable, on which the phonetic decision is made. In these two regions, the presence of noise consistently interferes with the categorization of the stimulus. When we match their time-frequency positions with the acoustical content of the stimuli (formant trajectories symbolized as lines in Fig 3), it appears that participants use two distinct acoustic cues for performing the task: namely the combined onsets of the 2^nd^ and 3^rd^ formants, and the onset of the 1^st^ formant. This observation is coherent with the literature [53,54], and reproduces the results of two previous ACI studies with the same Alda/Alga/Arda/Arga task performed by normotypic [33] or musician [34] listeners.

However this apparent similarity between the various groups of listeners can possibly mask more subtle differences in the listening strategies employed. A tempting explanation for the poor performances of dyslexic participants is that their phonological representations are somewhat noisy, or not precisely attuned to phonetic categories. This impairment might result here in a less efficient weighting of the acoustic cues in their ACIs than in the normotypical ones. Accordingly, we compared the ACIs for the dyslexic and control groups, using a cluster-based nonparametric test, but we didn’t detect any significant difference in ACIs of the two groups of listeners. Even a less conservative analysis showed that only one among 6 tested ROIs, corresponding to the central negative cue, was weighted differently in the two groups.

Nonetheless this null result could be due to the pattern being highly variable at the individual level. Indeed when looking more closely at the non-averaged ACIs Fig 3, we notice an important heterogeneity, especially in the dyslexic group, which may reflect specificities of each participant’s listening strategy, and/or a certain amount of noise due to the estimation process itself. In order to disentangle between these two possible causes, we calculated the individual auto- and cross-prediction deviances, a measure of the amount of error when predicting one participant’s data by his/her own ACI or by those of the others, respectively. The difference between the two predictions directly relates to the amount of the participant’s data accurately predicted only by his/her own ACI and mispredicted by the others. Therefore it is an indicator of how the estimated ACI reflects the specificities of the listener’s strategy.

Our rationale was the following: if the observed variability between the dyslexics’ ACIs was primarily due to the presence of individual strategies, as hypothesized earlier, the absolute difference between auto- and cross-prediction deviances should be higher in the dyslexic group than in the control group. On the contrary, if the ACIs in the dyslexic group are just less accurately estimated (due for example to the higher SNR in their stimuli), this would mainly impact the auto-prediction. In this case the absolute difference between auto- and cross-prediction deviances should be lower in the dyslexic group than in the control group.

The results showed that the measured cross-prediction deviance was always higher than the auto-prediction deviance (i.e. data points are above the dotted line on Fig 4A) indicating that the participants’ responses are better predicted by their own ACI, which captures some idiosyncratic aspects of the processing. Moreover the difference between auto- and cross-prediction deviances is larger in the dyslexic group than in the control group. This result is in line with our hypothesis that participants with dyslexia show individual, less efficient, weighting strategies in the task. However, according to Fig 4, this observation does not hold for the group as a whole but it is only due to a subgroup of 7 dyslexics with very high “specificity”. Indeed the distribution of weights inside their ACIs suggests a specific preference in this population for other auditory cues than those preferentially exploited by control participants. The most striking effect is observed for participant D15, whose ACI does not show the usual negative cluster of weights around 2500 Hz, nor the low-frequency cue. Consequently, his/her responses are poorly predicted by the other ACIs (cross-prediction deviance = 418.4). However the auto-prediction deviance within the normal range (= 273.7) assesses that this ACI can generalize to unseen data from participant D15, and therefore that it accurately models the underlying process. The same holds for participants D1, D5, D6, D7, D18 and D21, corresponding to the most scattered distributions of perceptual weights.

The finding that some dyslexics in our sample used individual listening strategies is coherent with the hypothesis that dyslexia is related to an impairment of phonological representation. However, the specificity measure is strongly correlated with the mean SNR at which participants performed the task. Furthermore we did not find any robust correlation with the tests in the cognitive and phonological battery, even after grouping the tests in 5 composite scores [7] reflecting phonological awareness (r(16) = 0.01, p = 0.95), literacy (r(16) = 0.02, p = 0.92), short term memory (r(16) = -0.44, p = 0.06), nonverbal IQ (r(16) = 0.35, p = 0.15), and attention (r(16) = -0.06, p = 0.82). Therefore, it is not clear from these results whether the strategy change in the dyslexic subgroup has caused their lower performances in the categorization task or, conversely, if the dissimilarities observed in their ACIs are a consequence of the gap in SNR. Indeed we have previously demonstrated that the level of noise influences the processing of stimuli [32] (it should be noted however that in this previous study we only evidenced a change in cue weighting, not in the strategy as it seems the case here). In order to clarify this issue, it would be very useful to compare the ACIs of the dyslexic group with those of a second control group, matched in reading-level rather than in chronological age, to ensure that observed differences could not be explained by noise level differences alone.

Finally, we excluded the subgroup of 7 lower-performing participants to explore in further detail the group differences between the ACIs of dyslexics and average readers completing the task with equivalent SNRs. Our hypothesis was that the 11 remaining high-performing dyslexics successfully developed compensatory strategies for their phonologic impairment, yielding normal recognition in the phoneme in noise categorization task. A new cluster-based comparison (N = 11) revealed that they indeed have a slightly different strategy than controls, relying more on the onset of F3 (and maybe F4) in the first syllable (Fig 5). To reach the same level of performances than their average reader peers, dyslexics seems to be using a slightly different listening strategy, involving the “classic” F1, F2 and F3 onsets from the syllable to be categorized, but also anticipatory cues.

The interpretation of these cues is not clear, however. They may correspond to subtle differences in the amplitude or timing of the onsets. According to the allophonic perception theory [15,16,30], dyslexic individuals demonstrate an excessive sensitivity to non-linguistic information in the acoustic signal. Therefore, they may be able to extract allophonic cues to build their compensatory strategy, relying on the redundancies in our speech targets. On the ACI these two cues appear as small but significant clusters of negative weights, suggesting that these cues may not be used for all trials. One possibility is that these secondary cues affect the decision only when the primary cues are ambiguous [55].

This finding that high-performing dyslexics used allophonic cues can be linked to a series of recent neuroimaging studies revealing that, even when dyslexics show normal behavioral responses in a speech-in-noise task [22], or in a phoneme categorization task [29,30], their deficit still manifest in the form of enhanced neurophysiological activity. This has been proposed by the authors as a demonstration of the less efficient strategies dyslexics employ to compensate for their impaired phonological processing. In the present study we showed that these strategies can be revealed by the purely behavioral ACI methodology. For our subgroup of 11 dyslexics performing as well as average readers in a phoneme categorization task in noise, the extraction of allophonic cues requires additional cognitive resources, potentially leading to increased neural activations and to stronger mental fatigue.

Two limitations of this study must be highlighted here. First, the last finding relies on a comparison between two subgroups of N = 11 participants, and therefore calls for a replication with a larger sample. Second, one general constraint of the ACI technique is the large amount of trials required for the estimation (10,000 categorizations per participant in the present study), and the limited number of targets. As a consequence, listeners might use stimulus-specific strategies for performing the task, which casts doubts on the authenticity of the acoustic cues revealed in this way. That does not seem to be the case for the control group, as their results are consistent with the literature. However one can ask whether the alternative strategy employed by dyslexic participants is not due to their difficulties in such long and repetitive tasks.

## Supporting Information

S1 Table Summary of the characteristics of the dyslexic and normal-reading subgroups. (DOCX)
