# Supplementary material for: Direct Viewing of Dyslexics’ Compensatory Strategies in Speech in Noise Using Auditory Classification Images
Source: PLoS One. 2016 Apr 21;11(4):e0153781. doi: 10.1371/journal.pone.0153781 (PMC4839691; doi:10.1371/journal.pone.0153781)
Supplement: S1 Table — (DOCX) [file pone.0153781.s001.docx]

Direct viewing of dyslexics’ compensatory strategies in speech in noise using auditory classification images. (Varnet, Meunier, Trollé & Hoen, 2016)

**Supplementary data**

|  |  | Dyslexic subgroup | Normal-hearing subgroup | t-test |
| --- | --- | --- | --- | --- |
| N |  | 11 | 11 |  |
| Gender (f/m) | | 6/5 | 8/3 |  |
| Age |  | 23.64 (± 7.45 S.D.) | 20.91 (± 1.22 S.D.) | p=0.24 |
| Handedness (Edinburgh test) | | 70.90 (± 35.90 S.D.) | 70.00 (± 46.04 S.D.) | p=0.95 |
| Raven’s Standard Progressive Matrices (score /60) | | 50.54 (± 5.08 S.D.) | 50.63 (± 4.01 S.D.) | P=0.96 |
| Reading age in months (L'Alouette MCLM) | | 126.27 (± 32.68 S.D.) | 187.27 (± 24.61 S.D.) | p=7.10^-5^*** |
| Reading tests | regular words (score /20) | 19.09 (± 0.83 S.D.) | 19.82 (± 0.40 S.D.) | p=0.016* |
|  | regular words (time in s) | 21 (± 8.79 S.D.) | 11.91 (± 2.02 S.D.) | p=3.10^-3^** |
|  | irregular words (score /20) | 18.27 (± 2.05 S.D.) | 19.09 (± 0.83 S.D.) | p=0.23 |
|  | irregular words (time in s) | 19.91 (± 8.0926 S.D.) | 11.73 (± 2.10 S.D.) | p=4.10^-3^** |
|  | pseudowords (score /20) | 16.91 (± 4.18 S.D.) | 19.18 (± 0.87 S.D.) | p=0.09 |
|  | pseudowords (time in s) | 35.09 (± 14.80 S.D.) | 18.55 (± 3.50 S.D.) | p=2.10^-3^** |
| Spelling tests | sentences: orthography (score /10) | 6.18 (± 1.89 S.D.) | 9.45 (± 0.52 S.D.) | p=2.10^-5^*** |
|  | sentences: grammar (s/10) | 6.45 (± 2.30 S.D.) | 8.82 (± 0.75 S.D.) | p=4.10^-3^** |
|  | regular words (score /10) | 6.82 (± 1.40 S.D.) | 9.18 (± 0.75 S.D.) | p=8.10^-5^*** |
|  | regular words (time in s) | 48.00 (± 7.24 S.D.) | 40.00 (± 6.16 S.D.) | p=0.01* |
|  | irregular words (score /10) | 4.09 (± 2.21 S.D.) | 7.82 (± 1.40 S.D.) | p=1.10^-4^*** |
|  | irregular words (time in s) | 51.18 (± 11.19 S.D.) | 43.36 (± 6.30 S.D.) | p=0.06 |
|  | pseudowords (score /10) | 7.55 (± 1.04 S.D.) | 9.00 (± 1.26 S.D.) | p=7.10^-3^** |
|  | pseudowords (time in s) | 56.45 (± 13.49 S.D.) | 44.91 (± 5.26 S.D.) | p=0.055 |
| Phonological awareness tests | Phoneme deletion (score /10) | 7.09 (± 2.34 S.D.) | 9.45 (± 1.51 S.D.) | p=0.011* |
|  | Phoneme deletion (time in s) | 49.00 (± 13.38 S.D.) | 28.73 (± 7.50 S.D.) | p=2.10^-4^*** |
|  | Spoonerism (score /20) | 15.00 (± 5.31 S.D.) | 19.18 (± 1.17 S.D.) | p=0.019 |
|  | Spoonerism (time in s) | 141.36 (± 64.53 S.D.) | 68.27 (± 22.55 S.D.) | p=2.10^-3^ |
| Memory span tests | Pseudowords repetition (score /20) | 19.18 (± 1.08 S.D.) | 19.55 (± 0.69 S.D.) | p=0.36 |
|  | Forward digit | 6.36 (± 0.67 S.D.) | 7.36 (± 1.12 S.D.) | p=0.02 |
|  | Backward digit | 4.73 (± 1.49 S.D.) | 6.18 (± 1.33 S.D.) | p=0.025 |
| ANT | alerting effect | 30.82 (± 23.30 S.D.) | 36.36 (± 22.46 S.D.) | p=0.58 |
|  | orienting effect | 52.55 (± 14.87 S.D.) | 36.09 (± 24.69 S.D.) | p=0.073 |
|  | conflict effect | 147.00 (± 39.53 S.D.) | 141.18 (± 41.77 S.D.) | p=0.74 |

**Table S1. Summary of the characteristics of the dyslexic and normal-reading subgroups**
